# Comparison of myocardial perfusion scintigraphy and strain echocardiography in patients undergoing coronary angiography

**DOI:** 10.1590/1806-9282.20241806

**Published:** 2025-05-02

**Authors:** Çağlar Kaya, Muhammet Gürdoğan

**Affiliations:** 1 Trakya University, Department of Cardiology – Edirne, Turkey.

**Keywords:** Two-dimensional echocardiography, Global longitudinal strain, Myocardial perfusion imaging

## Abstract

**OBJECTIVE:**

Myocardial perfusion scintigraphy is a common non-invasive method for assessing ischemic burden, though artifacts can affect accuracy. Speckle-tracking strain echocardiography improves left ventricular function assessment, and global longitudinal strain correlates well with coronary artery disease. The aim of this study was to compare myocardial perfusion scintigraphy with global longitudinal strain in stable angina pectoris patients.

**METHODS:**

A total of 133 suspected coronary artery disease patients who underwent myocardial perfusion scintigraphy and coronary angiography were prospectively enrolled and classified as myocardial perfusion scintigraphy true positives or false positives based on coronary angiography results. Global longitudinal strain values for the epicardium, endocardium, and myocardium (avg) were calculated.

**RESULTS:**

Ischemic percentages of myocardial perfusion scintigraphy>12% and mid-wall global longitudinal strain<-18.4% correlated with true positive coronary angiography results. Left ventricular ejection fraction/global longitudinal strain mid ratio positively correlated with coronary artery disease presence and severity. Higher ischemic percentages of myocardial perfusion scintigraphy showed a negative correlation (r: −0.2606, p: 0.002) with global longitudinal strain, indicating a greater likelihood of coronary artery disease (OR 0.25, 95%CI 0.08–0.73, p: 0.012). Female sex was linked to fewer true positive myocardial perfusion scintigraphy results.

**CONCLUSION:**

The GLS value of the Left Ventricle obtained by two-dimentional strain echocardiography offers sensitivity and specificity similar to myocardial perfusion scintigraphy in the detection of coronary artery disease.. An elevated left ventricular ejection fraction/global longitudinal strain ratio is a significant predictor of the presence and severity of coronary artery disease.

## INTRODUCTION

Coronary artery disease (CAD) is a major cause of global mortality. Stable angina pectoris (SAP), or chronic coronary syndrome, is a specific form of CAD. Non-invasive diagnostic methods are preferred for evaluating patients with SAP^1^.

Myocardial perfusion scintigraphy (MPS) is a non-invasive imaging technique with high accuracy for evaluating ischemic burden in SAP. It identifies ischemic and infarcted areas after radiopharmaceutical injection. However, MPS is susceptible to artifacts and challenges that can lead to false-positive (FP) or false-negative CAD assessments.

Two-dimensional (2D) transthoracic echocardiography is a common non-invasive method for detecting SAP, providing important information on left ventricular ejection fraction (LVEF) and wall motion. Adding speckle tracking to echocardiography enhances the detection of CAD by allowing precise assessments of segmental myocardial deformation^2^. Longitudinal myocardial fibers are the most sensitive to ischemia and are predominantly located in the subendocardial layer. According to the literature, there is a strong correlation between myocardial deformation values obtained by global longitudinal strain (GLS) analysis and the presence of CAD.

This study aimed to compare MPS findings with GLS values derived from strain echocardiography (S-ECHO) in patients with SAP who underwent coronary angiography (CAG).

## METHODS

Between July 2023 and August 2024, 133 patients who underwent MPS and CAG with suspected CAD were prospectively included in the study. Patients with acute coronary syndrome, a history of revascularization (stent and bypass), heart failure with low ejection fraction (EF), and moderate-to-advanced valvular diseases were excluded. The patients were divided into two main groups according to the results of CAG: MPS true positives (MPS-TPs) and MPS -FPs. Percentages and regions in MPS were also recorded. Patients who underwent CAG due to MPS positivity were included. The study was conducted in compliance with the Declaration of Helsinki. The study protocol was approved by the Institutional Committee on Human Research and Ethics. All patients provided a written informed consent. The Trakya University's Ethics Committee approved this study (clinical trial approval number: TÜTF-GOBAEK-2023/398).

### Data assessments

Echocardiographic images were obtained using Vivid S70 systems (GE Healthcare, Horton, Norway) and were analyzed on the Echo-PAC workstation. Conventional echocardiographic measurements followed standard guidelines, with the software automatically calculating GLS values for epicardial, endocardial, and myocardial layers (GLS-epi, GLS-endo, and GLS-mid [avg], respectively), with GLS-mid recorded as GLS Avg. Severe CAD was defined by CAG ≥70% stenosis, and SYNTAX scores were calculated for these cases using the online tool (version 2.28)^3^. After the SYNTAX scores were calculated, the patients were divided into three groups as described in the literature^4^. This grouping was made as SYNTAX scores 0–22, 23–32, and 33 and higher. Patients with severe CAD were placed in the MPS TP group.

### Statistical analysis

Statistical analyses were conducted using SPSS version 25.0 (SPSS, Chicago, IL, USA). Continuous variables were presented as means and standard deviations, while categorical variables were reported as counts and percentages. The Shapiro-Wilk test assessed normality. An independent samples t-test compared continuous variables between two groups, and the Pearson's chi-square test evaluated relationships among categorical variables. Receiver operating characteristic curve analysis determined curve areas and cutoff points. Logistic regression analysis identified independent variables. All tests were two-tailed, with p<0.05 deemed statistically significant.

## RESULTS

In this study, a total of 133 patients were enrolled and divided into two groups: 58 (43.60%) in the MPS-FP group and 75 (56.40%) in the MPS-TP group. The MPS-FP group showed a female predominance (60.35%, p=0.002). The mean age of all patients was 59 years, with the MPS-TP group being older. Higher rates of diabetes, hypertension, and smoking were observed in the MPS-TP group, as expected. Conventional echocardiographic parameters, including LVEF, left ventricular (LV) diameter, and LV mass index, did not differ significantly. The MPS percentages were 14.88±5.28 for the MPS-TP group and 11.10±2.79 for the MPS-FP group. Table 1 presents the clinical characteristics and echocardiographic parameters of the study population comparing the MPS-TP and MPS-FP groups.

**Table 1:** The clinical characteristics of patients and some conventional echocardiographic and two-dimensional global longitudinal strain parameters of the study population in comparison with myocardial perfusion scintigraphy true positives and myocardial perfusion scintigraphy false positives.

Variables	MPS false positives (NS-CAD) (n=58) (mean±SD)	MPS true positives (CAD, yes) (n=75) (mean±SD)	p
Age (years)	55.94±9.99	61.70±9.61	<0.001
Gender			
Male, n (%)	23 (39.65)	50 (66.67)	
Female, n (%)	35 (60.35)	25 (33.33)	0.002
BMI, kg/m^2^	29.47±3.84	29.21±4.64	0.728
DM, n (%)	12 (20.68)	32 (42.66)	0.008
HT, n (%)	48 (82.75)	63 (84)	0.848
Smoking, n (%)	23 (39.65)	43 (57.33)	0.036
HL, n (%)	24 (41.37)	49 (65.33)	0.006
LVEF (%)	65.75±4.47	64.73±5.37	0.251
E/e’	8.36±2.27	9.11±2.23	0.029
LVEDD (mm)	46.98±3.88	47.12±4.18	0.847
LVESD (mm)	29.53±3.43	30.24±3.82	0.272
LV mass index (g/m^2^)	97.02±16.41	101.81±22.45	0.175
LA volume index (mL/m^2^)	21.64±4.58	21.83±5.33	0.761
Percentage of ischemia in MPS	11.10±2.79	14.88±5.28	<0.001
GLS mid-myo (avg), %	-22.04±2.14	-15.48±2.37	<0.001
GLS endocardial, %	-24.99±2.48	-18.43±2.95	<0.001
GLS epicardial, %	-19.53±1.80	-14.92±2.60	<0.001
LVEF/GLS Avg ratio	3.05±0.32	3.86±0.61	<0.001

BMI: body mass index; CAD: coronary artery disease; Cx: circumflex artery; DM: diabetes mellitus; HL: hyperlipidemia; HT: hypertension; EDD: end-diastolic diameter; EF: ejection fraction; ESD: end-systolic diameter; LA: left atrial; LV: left ventricular; MPS: myocardial perfusion scintigraphy; NS: non-significant; GLS: global longitudinal strain; LVEF: left ventricular ejection fraction; Avg: average.

Vascular disease distributions were analyzed by the vessel count and lesion location: 27 patients (36.0%) had single-vessel disease, 18 (24%) had two-vessel disease, and 30 (40%) had three-vessel disease. Left anterior descending (LAD) lesions were most common (81.33%), with circumflex artery (CX) and right coronary artery (RCA) lesions showing similar rates (60 and 61.33%, respectively). All GLS values were statistically significantly lower in the MPS-TP group (p<0.001) (Table 1). Ischemia was mainly in the anterior (68%) and lateral (52%) regions, followed by inferior (47%), septal (26%), and apical (14%) regions. In the MPS-TP group, Pearson's correlation coefficient revealed a negative correlation between MPS ischemic percentages and GLS values (Table 2).

**Table 2:** Diagnostic performance of myocardial perfusion scintigraphy percentage: Global longitudinal strain parameters and the correlation between ischemic percentages of myocardial perfusion scintigraphy and global longitudinal strain values in the myocardial perfusion scintigraphy true-positive group.

Diagnostic performance of GLS parameters in the true-positive group							
	Cutoff	AUC (95%CI)	Sensitivity		Specificity		p
GLS (Avg)	-18.4	0.90 (0.84–0.95)	0.74 (0.63–0.83)		0.96 (0.88–0.99)		<0.001
GLS endocardial	-22.3	0.91 (0.84–0.95)	0.84 (0.74–0.90)		0.86 (0.75–0.92)		<0.001
GLS epicardial	-16.4	0.90 (0.83–0.94)	0.77 (0.66–0.85)		0.96 (0.88–0.99)		<0.001
Diagnostic performance of MPS percentages in the true-positive group							
		Cutoff	AUC (95%CI)			p	
Percentage of ischemia in MPS		>12	0.76 (0.68–0.86)			<0.001	
Sensitivity: 0.77 (0.66–0.85)			Specificity: 0.81 (0.69–0.89)				
Correlation between ischemic percentages of MPS and GLS values in the true-positive group							
		Correlation (r)		p			
GLS (Avg)		-0.2606		0.002			
GLS endocardium		-0.2716		0.002			
GLS epicardium		-0.2636		0.002			

GLS: global longitudinal strain; Avg: average; MPS: myocardial perfusion scintigraphy; AUC: area under the curve; CI: confidence interval.

While the diagnostic performance of MPS percentages and GLS parameters in the MPS-TP group was evaluated, the GLS mid (avg) cutoff value for predicting CAD in the MPS-TP group was <-18.4. Sensitivities and specificities were estimated to be in the range of 84–86% (area under the curve [AUC]: 0.91, p<0.001). When the cutoff values for MPS were analyzed, a value of 12% was found (Table 2).

Correlation analysis showed a statistically significant increase in the LVEF/GLS ratio with higher SYNTAX scores (r=0.3164, p=0.006). While GLS values did not differ statistically significantly by the number of affected vessels (p=0.315), the LVEF/GLS ratio was statistically significantly higher in three-vessel disease than in single-vessel disease (3.69±0.56 vs. 4.07±0.45, p=0.042). By SYNTAX grouping, a decrease in GLS values and an increase in the LVEF/GLS ratio were observed (p=0.038). The SYNTAX scores showed a statistically significant negative correlation with all GLS values (GLS Avg: r =-0.3361, p=0.003). Regression analysis identified age, male sex, and GLS mid (mean) as independent variables (Table 3). In a multivariate model, the LVEF/GLS ratio was also found as an independent risk factor (OR 231, p<0.001).

**Table 3:** Uni-multivariate and stepwise binary logistic regression analyses for myocardial perfusion scintigraphy-TPs.

Log reg	Univariate model-1					Multivariate model-1			
	OR		95%CI	p		OR	95%CI		p
Age	1.062		1.02–1.10	0.002		1.12	1.02–1.23		0.018
Sex (male)	3.043		1.49–6.20	0.002		12.9	1.60–104.9		0.016
DM	2.853		1.30–6.24	0.009		2.00	0.33–12.07		0.446
Smoking	2.045		1.01–1.10	0.044		0.97	0.18–5.17		0.977
LVEF/GLS	103.0		20–514	<0.001		2.34	0.01–1836		0.802
GLS-endo	0.441		0.34–0.58	<0.001		0.67	0.30–1.51		0.345
GLS-avg	0.381		0.27–0.53	<0.001		0.25	0.08–0.73		0.012
GLS-epi	0.339		0.23–0.49	<0.001		1.79	0.57–5.67		0.217
EF	0.961		0.89–1.02	0.250		1.22	0.98–1.51		0.067
Multivariate model-2									
		OR			95%CI			p	
Age		1.17			1.06–1.29			0.002	
Sex (male)		5.57			1.06–29.2			0.042	
DM		0.89			0.19–4.08			0.886	
Smoking		0.95			0.19–4.65			0.956	
LVEF/GLS		231			24–2222			<0.001	

Log Reg: logistic regression; GLS: global longitudinal strain; Avg: average; Epi: epicardium; Endo: endocardium; EF: ejection fraction; DM: diabetes mellitus; CI: confidence interval; OR: odds ratio.

In the analyses performed for lesion prediction, a decrease in regional longitudinal strain (RLS) values related to the lesion site was found for LAD and RCA lesions. In patients with CX lesions, there was a statistically significant decrease in RLS values related to both the LAD and CX territories (p<0.001).

## DISCUSSION

The main findings of the study can be summarized as follows: (1) In patients with SAP, there was a correlation between MPS results above 12% (sensitivity: 0.77, specificity: 0.81) and mid-wall GLS (avg) values below −18.4% (sensitivity: 0.74, specificity: 0.96) with TP CAG outcomes. (2) Within the MPS-TP group, there was a positive correlation between the LVEF/GLS mid ratio and both the presence and severity of CAD. (3) A negative correlation was identified between increasing ischemic percentages of MPS and GLS values, indicating that this correlation is associated with an increased likelihood of TP CAD. (4) Additionally, it was found that female sex was associated with a reduced rate of TP results in MPS.

Risk stratification in SAP is essential for optimal management. The recent European Society of Cardiology guidelines recommend direct invasive CAG for patients with a pre-test probability of CAD over 85%. For those with a pre-test probability between 15 and 85%, non-invasive stress testing, preferably with imaging, is advised to reduce costs and complications following invasive procedures^1^. Although treadmill or bicycle exercise testing is cost-effective, safe, and widely available, it has been used less frequently in recent years because of its low sensitivity^5^. MPS is recommended by clinical guidelines for assessing ischemic burden in patients with suspected angina pectoris and a moderate-to-high pre-test probability (15–85%). Additionally, it is essential for risk stratification and treatment planning in patients diagnosed with CAD^1^. In patients with angina symptoms, the MPS method has a sensitivity of 86–88% and a specificity of 74–76% for detecting coronary stenosis^6^. While this method is valuable for diagnosing CAD, its main limitation is the radiation exposure from thallium-201 radiopharmaceuticals. Additionally, limited availability in various health-care settings and the need for skilled specialists to interpret results pose further challenges^6^. MPS has a significant rate of false-negative and FP results^7^. It may not accurately reflect CAD severity in patients with multivessel disease. Factors contributing to FPs, especially in obese individuals and women, include attenuation artifacts, technical limitations, coronary vasospasm, circulation abnormalities, cardiomyopathy, and conduction issues like left bundle branch block^8^. The guidelines recommend that CAG should be considered if MPS reveals ischemia of 10% or more^1^. In our study, we found an ischemic burden of 12% as a cutoff value for MPS in the detection of CAD in the MPS-TP group. Therefore, additional imaging techniques are required to guide the invasive strategy in distinguishing between these closely related values.

Echocardiography is a widely used, radiation-free technique. However, suboptimal image quality in some patients can hinder the assessment of segmental motion in CAD, and an accurate visual analysis requires an experienced operator^9^. Conventional echocardiographic measurements can obscure regional wall motion abnormalities, but studies show that 2D speckle-tracking strain analysis can reveal subtle myocardial changes indicative of CAD^10^. In our study, we identified a threshold of <-18.4% for LV/GLS (avg) to detect CAD in the MPS-TP group using S-ECHO. The literature suggests that S-ECHO provides valuable information not only in the detection of CAD but also in the assessment of wall motion abnormalities^11^. In the literature, a study examining 268 patients with negative cardiac biomarkers and non-specific electrocardiographic (ECG) findings but with a suspicion of CAD identified a cutoff value of −18.8% for LV/GLS. In their study, the sensitivity of GLS was found to be 72%, while the specificity was 82%^12^. In our study, the cutoff value for LV/GLS (avg) was determined to be −18.4%, with a sensitivity of 74% and a specificity of 96% for GLS. Furthermore, a decrease in LV/GLS values was confirmed to be an independent predictor of stable CAD in patients without wall motion abnormalities^10^. On the other hand, the literature suggests that negative results for LV/GLS obtained by S-ECHO may provide an acceptable negative predictive value for CAD^9^. In our study, we similarly found that GLS (avg) measurements serve as a negative predictor of CAD for patients with TP results in MPS. It has been determined that S-ECHO can guide the discharge of patients with suspected CAD who have normal GLS values from emergency departments or cardiology clinics.

S-ECHO has several advantages over MPS, including enhanced accessibility, greater repeatability, and shorter procedure times^13^. The literature shows that patients with CAD have lower left ventricular GLS values than those with normal coronary arteries. Additionally, patients with significant stenosis in a specific artery exhibit a greater reduction in RLS from that segment. Thus, S-ECHO is useful for diagnosing CAD and identifying the responsible lesions in affected coronary arteries^14^. However, in our study, while the subgroup analysis of patients with MPS-TPs revealed that the endo-mid-epi values of LAD-RLS from the same segment were lower compared to other regions (with LAD-lesion −16.51; without −20.64, p<0.001), regression analysis indicated that no RLS value served as an independent predictor for predicting LAD lesions (OR 3.35, 95%CI 0.16–67.23, p:0.408). Similarly, the literature demonstrates that studies utilizing speckle-tracking echocardiography in patients with total and subtotal stenosis in different coronary arteries have successfully distinguished between the degrees of stenosis^15^. In our study, although a greater reduction was observed in RLS values applied for lesion-specific evaluation, we determined that none of the RLS parameters could be utilized as predictors for lesions (p>0.05). The most likely reasons for this finding may include the fact that coronary arteries do not solely supply specific segments, variations during the coronary arteries, the presence of collateral circulation, and microvascular circulation disturbances.

A remarkable finding of our study was the increase in the LVEF/GLS ratio in MPS-TP patients, independent of EF. We observed that as the SYNTAX score increased, GLS values decreased and the LVEF/GLS ratio rose, indicating a negative correlation. This suggests that the LVEF/GLS ratio may indicate disease prevalence beyond just CAD presence. In a study conducted on acute coronary syndrome patients in the literature, GRACE score, SYNTAX score, and GLS values were used. In this study, it was found that the LV/GLS ratio could be used in patients with high GRACE scores^16^. Therefore, we believe that the increase in the LVEF/GLS ratio in patients with stable CAD as seen in our study can be used to predict subclinical LV dysfunction. It is known that the specificity and sensitivity of MPS are lower, and its diagnostic value is reduced in women. Possible reasons for this are attenuation artifacts or anterior chest wall morphologies^8^. Breast tissue most commonly causes artifacts on the anterior wall. In other regions, the diaphragm in the inferior wall and adipose tissue in the lateral wall may cause artifacts. In our study, we observed a female predominance in the MPS-FP group. LVEF/GLS examination may be an important alternative to MPS in female patients with SAP and CAD.

## CONCLUSION

In SAP patients, LV/GLS from 2D speckle-tracking echocardiography shows sensitivity and specificity similar to MPS for detecting CAD. A higher LVEF/GLS ratio is a strong indicator of CAD severity. LV/GLS is a useful non-invasive option, especially for female patients prone to MPS FPs due to artifacts.

## Data Availability

The data that support the findings of this study are available from the corresponding author upon reasonable request.
